# Identifying drivers of increasing opioid overdose deaths among black individuals: a qualitative model drawing on experience of peers and community health workers

**DOI:** 10.1186/s12954-023-00734-9

**Published:** 2023-01-13

**Authors:** Devin E. Banks, Alex Duello, Maria E. Paschke, Sheila R. Grigsby, Rachel P. Winograd

**Affiliations:** 1 Department of Psychological Sciences, University of Missouri—St. Louis, One University Blvd., 325 Stadler Hall, St. Louis, MO USA; 2 Missouri Institute of Mental Health, University of Missouri—St. Louis, One University Blvd., St. Louis, MO USA; 3 College of Nursing, University of Missouri—St. Louis, One University Blvd., St. Louis, MO USA

**Keywords:** Black Americans, Racial disparities, Opioids, Overdose, Racism, Substance use treatment, Community-based participatory research

## Abstract

**Background:**

Black individuals in the USA face disproportionate increases in rates of fatal opioid overdose despite federal efforts to mitigate the opioid crisis. The aim of this study was to examine what drives increases in opioid overdose death among Black Americans based on the experience of key stakeholders.

**Methods:**

Focus groups were conducted with stakeholders providing substance use prevention services in Black communities in St. Louis, MO (*n* = 14). One focus group included peer advocates and volunteers conducting outreach-based services and one included active community health workers. Focus groups were held at community partner organizations familiar to participants. Data collection was facilitated by an interview guide with open-ended prompts. Focus groups were audio recorded and professionally transcribed. Transcripts were analyzed using grounded theory to abstract line-by-line codes into higher order themes and interpret their associations.

**Results:**

A core theme was identified from participants’ narratives suggesting that opioid overdose death among Black individuals is driven by unmet needs for safety, security, stability, and survival (The 4Ss). A lack of The 4Ss was reflective of structural disinvestment and healthcare and social service barriers perpetuated by systemic racism. Participants unmet 4S needs are associated with health and social consequences that perpetuate overdose and detrimentally impact recovery efforts. Participants identified cultural and relationship-based strategies that may address The 4Ss and mitigate overdose in Black communities.

**Conclusions:**

Key stakeholders working in local communities to address racial inequities in opioid overdose highlighted the importance of upstream interventions that promote basic socioeconomic needs. Local outreach efforts utilizing peer services can provide culturally congruent interventions and promote harm reduction in Black communities traditionally underserved by US health and social systems.

## Background

Despite increased federal attention and funding over the last decade, the opioid overdose crisis poses an unrelenting public health threat in the USA. Drug overdose deaths reached an annual record high of more than 100,000 in April 2021, an increase of nearly 30% from the US’s previous record high in 2020 [1]. Driven by opioid overdose deaths (OOD), these record highs are especially concerning for Black Americans, who outpaced other racial/ethnic groups in year-over-year increases in OOD from 2014 to 2019 [2]. Considering concurrent decreases among white Americans during that time, in 2021, the National Institute on Drug Abuse concluded that “Black individuals have not benefited equally from prevention and treatment efforts” aimed at the opioid crisis during the last decade [3].

Racial inequities in the growth rate of OOD have been documented in several metropolitan locales and at least 23 states including Missouri, the state with the second highest Black–white disparity in drug overdose death and the second highest rate of OOD among Black individuals [4, 5]. These rates are driven by the St. Louis region, which accounted for 78% of drug overdose deaths among Black individuals from 2020 to 2021, despite only 54% of Missouri’s Black population residing in this region [6]. Like in other regions, Black OOD in St. Louis have outpaced white OOD since the introduction of illicitly manufactured fentanyl and its analogues into Missouri’s drug supply in 2016 [7, 8].

In addition to high rates of OOD, St. Louis has one of the lowest ratios of opioid use disorder (OUD) treatment capacity to OOD in the USA [9]. Due to this gap in treatment access, St. Louisans are building a robust workforce of peer- and community health workers in both community and clinical settings. Peer support services employ individuals with first-hand lived experience with substance use disorder, whereas community health workers (CHWs) have first-hand lived experience in the communities they serve; both groups often share similar racial and demographic backgrounds to their clients [10]. Outreach-based interventions dispatching peers and CHWs show promising rates of linkage to and engagement with substance use treatment and social services [11–14]. These interventions are particularly important for improving outcomes among Black people who use drugs (PWUD) as they increase cultural relevance and trust in service provision [12, 14].

The current study leverages the local knowledge of key stakeholders conducting outreach in Black communities (i.e., peers and CHWs) to examine their perceptions of why OOD has increased disproportionately among Black people in St. Louis (588% relative to 48% among whites from 2015 to 2021). Although this research is urgent in St. Louis, findings have implications for regions that face similar inequities in OOD. Our approach, grounded in community-based participatory research, honors the ongoing work community members are undertaking to address the overdose crisis in Black communities. Using grounded theory to develop a comprehensive model, this approach will help identify practical solutions that reduce the burden of OOD among Black PWUD.

## Methods

### Sample

Data from these focus groups were derived as part of the CENTER (Community ENgagement, Trauma, Equity, and Renewal) Initiative (centerstl.org), an academic–community partnership aimed to reduce overdose, confront the impact of trauma, and invest in the long-term well-being of Black people most impacted by addiction, drug use, and overdose in St. Louis. Researchers worked closely with community partners throughout project development and implementation. Partners were selected based on experience working toward health equity in Black communities and included several social service nonprofits and grassroots healthcare organizations. To recruit participants, community partners received a short description of the focus groups and recruited participants via word of mouth. Recruitment followed a purposive or “expert” sampling method consistent with grounded theory principles [15, 16]. The first focus group included peer advocates and volunteers conducting street outreach at a local nonprofit (*n* = 7). The second included active CHWs (*n* = 7). Most participants were Black (*n* = 11; 79%) and provided both professional and personal perspectives on drivers of OOD.

### Procedures

Interview guides used open-ended prompts aimed at exploring factors underlying OOD among Black PWUD. Prompts explored quality of substance use treatment services, changes in the drug supply, perceptions of harm reduction practices, and needed services for Black PWUD. Aligned with purposive sampling and constant comparison, the line of questioning evolved between the first and second focus group [16]. One follow-up interview with two participants from the first group was conducted to improve understanding of several themes.

Focus groups took place in 2021 at local nonprofits familiar to the participants. Although not present during the focus groups, community partners who helped recruit participants greeted them and provided warm handoffs to research staff. Staff then met individually with each participant for informed consent procedures. Before each group began, participants discussed and agreed upon conversation and “safe space” expectations. Groups were audio recorded and lasted approximately 90 min. Participants were provided snacks and beverages and received $50 Visa gift cards for their participation. The University of Missouri—St. Louis Institutional Review Board, approved procedures.

The project was designed and overseen by the first author (DB), a Black woman and licensed clinical psychologist specializing in racial inequities in substance use. The second (AD) and third author (MP), who took notes during and facilitated focus groups, respectively, are public health professionals, each with over five years of experience in behavioral health service and substance use research. The fourth (SG) and fifth author (RW), who consulted on design and interpretation and approved all research materials, are scientist-practitioners bringing expertise in qualitative health disparities research and opioid overdose prevention and services, respectively.

### Data analysis

Audio files were transcribed verbatim by a professional agency, checked for accuracy by research staff, and uploaded to ATLAS.ti (Version 9) for analysis [17]. Researchers used a constant comparative method to collect data, document thoughts on data via memos, and develop meaning (i.e., codes). Data were analyzed by the first three authors in accordance with objectivist grounded theory in three stages [16, 18]: open, axial, and selective coding. Open codes were derived inductively from line-by-line coding to compare similar data events. This initial coding was reviewed by all authors and community advisory board (CAB) members, eight self-identified Black St. Louisans with lived experience with addiction. With CAB feedback, the research team developed a coding paradigm for abstracting open codes into higher-order axial codes and investigating the relationships between categories of open codes [15, 16]. Adapting Strauss and Corbin’s [15] coding paradigm, these axes concerned (1) causal conditions, (2) contextual conditions, (3) consequences, (4) strategies, and (5) intervening conditions. Open codes were abstracted into these axial categories based on their relations to several commonly coded phenomena. Through this iterative coding process, a core phenomenon emerged. In selective coding, this core phenomenon and its relation to the other axes became the focus of analysis until categories were saturated (i.e., additional data collection and coding no longer led to new insights) [16, 18]. The grounded theory that emerged from this process was shared with community partners, participants, and CAB members, who informed and confirmed saturation. Their feedback on the model was integrated into the following descriptive interpretation of the results, which is organized according to the coding paradigm.

## Results

In sum, the current grounded theory suggest Black OOD is driven by a lack of and/or need for basic safety, security, stability, and survival (hereafter abbreviated as The 4Ss; the core phenomenon), including inequitable access to resources associated with financial capital, lack of social support, and historical and ongoing racial and interpersonal trauma. Participants emphasized that many Black PWUD lack needed social determinants of health, including nutritious food, clean air, and safe living conditions, instead struggling to meet their basic needs day-to-day. Both groups also emphasized the lack of “safe space” or “safe haven(s)” for Black PWUD, where they could “have someone to talk to” and “be treated like a human being.” The scarcity of social capital (i.e., social resources and communal networks) was highlighted at the level and community level, with one participant stating, “I think part of [the reason Black people use drugs] is that we [Black people] lost the village. So now people are just trying to find ways…to cope and to deal with life.” This scarcity of both financial and social capital led to and exacerbated interpersonal and racial trauma, including early life adversity, further perpetuating The 4Ss and OOD.

**Fig. 1:**
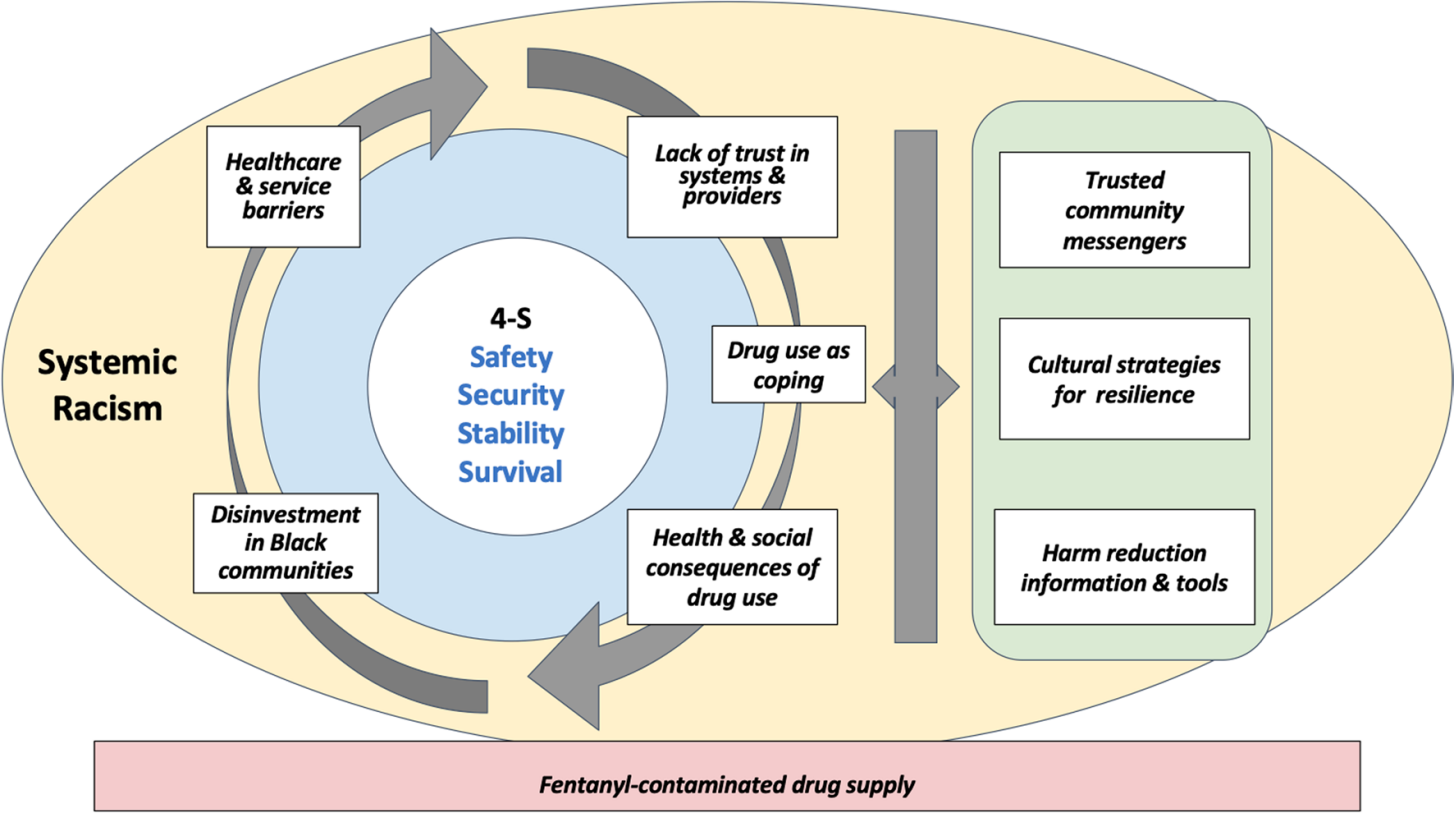
A grounded theory explaining drivers of opioid overdose among Black St. Louisans. *Note* arrows are meant to represent the interrelatedness of concepts rather than directionality

The following sections detail each theme of the grounded theory and their links to The 4Ss (Fig. 1), including illustrative (but not comprehensive) quotes. Specifically, participants described how Black St. Louisans were disproportionately exposed to The 4Ss due to systemic racism (i.e., the causal condition) on Black communities through structural disinvestment and on Black bodies through healthcare and social service barriers (i.e., contextual conditions). Participants associated a lack of fundamental 4S needs with health and social consequences that perpetuate drug misuse and treatment barriers (i.e., consequences), which in turn increased overdose risk. In response to these consequences, Black communities have developed cultural and relationship-based strategies to address The 4Ss and mitigate overdose in their own communities. Intervening conditions worsening The 4Ss and their impact on OOD include COVID-19 and the fentanyl-contaminated drug supply.

### Systemic racism

Participants pointed to systemic racism, historical and ongoing, as the causal condition driving a lack of 4Ss and ultimately, OOD among Black St. Louisans. Specifically, participants discussed four interrelated ways systemic racism manifests in disinvestment that impacts OOD among Black people: (1) racially inequitable funding systems, (2) resource deserts in Black communities, (3) lack of opportunities to build social capital and engage in healthy, adaptive activities, and (4) the over criminalization of Black communities, particularly drug criminalization. They also highlighted four ways systemic racism manifests in healthcare and service barriers: (1) poor perceived treatment quality, (2) lack of service capacity, (3) prohibitive cost of care, and (4) prohibitive eligibility/intake criteria.

#### Disinvestment in Black communities

Participants described how funding and resources for substance use treatment and social services are often not available for, or intentionally directed away from, Black neighborhoods and communities. They compared this lack of funding and practical resources to a perceived abundance of those in predominantly white neighborhoods:

If you go to the suburbs of St. Louis, Missouri, the rural areas…, they offering all different types of help when it comes to methamphetamine use…But when you come to the city, the inner city of St. Louis, Missouri, you get backlash. You get a whole bunch of excuses: "We don't have the facility. We don't have the capacity. We don't have the money. We don't have the funds. It's not available. We're full. See if you can come back." Who's to say that person is not dead by next week?

Participants also discussed how funding supposedly allocated to Black communities and OOD was improperly spent due to a lack of attention to or understanding of Black people’s most pressing needs: “We invest money in stuff that's really not too much important for the community, but then actual needs of the community, a lot of them are being overlooked.” Overlooked needs included shelters and stable housing, healthcare, transportation, and social services, which participants did not see expanding in their neighborhoods, citing misallocated or misdirected funding.

Relatedly, participants highlighted how systemic racism led to a lack of proximal opportunities in their environment to engage in healthy and adaptive activities, citing the overabundance of gas stations and liquor stores in their neighborhoods and a lack of community outlets like parks and community centers. Some described how being exposed to opportunities outside of their own neighborhoods was protective against drug use or overdose whereas other associated a lack of opportunity to escape neighborhood disadvantage with OOD risk: “What about when you do go to treatment, you got to return to this same neighborhood…You can't even try to change your scenery, so you just go back to using.”

Whereas systemic racism manifested in disinvestment in adaptive resources, it manifested in *over-investment* in the criminal-legal system in Black neighborhoods. Participants discussed a myriad of examples related to the over-investment of criminal-legal resources in Black neighborhoods, including victims of violence and overdose receiving suspicion rather than assistance from emergency responders. Participants were concerned about over-investment in drug criminalization among Black people specifically: “When a Black man get caught selling dope, he get life in prison. When a white man bring it over and get caught on the plane, he gets six month’s probation.”

#### Healthcare and service barriers

Systemic racism was also associated with barriers to accessing behavioral health services, substance use treatment, and other needed social services. Participants mostly did not distinguish between services, instead discussing how barriers applied across settings. Participants discussed poor treatment and care quality for Black PWUD not only in substance use treatment settings, but across healthcare and hospital settings, social services, housing, and law enforcement.

You get taken to [Hospital] or any other major ER system in the city. Black people especially are treated like they’re not humans. It’s like, despicable when I hear some of the stuff. No follow-up appointments, things like morphine drips or fentanyl drips for a month, full well knowing and understanding the problem that [opioid] dependency has in the Black community in St. Louis.

Participants emphasized the low capacity of service providers, specifically with regard to the lack of available “beds” to connect their clients to inpatient substance use treatment:

We have people tell us on the regular, "I'm ready to go [to treatment]." What can I tell them? There's no place to go. I can get you on a waiting list for if a bed becomes available. And then they back off, using again.

The lack of service availability was associated with perceived discriminatory treatment toward Black PWUD. Finally, prohibitive cost of treatment, including medication assisted treatment, and prohibitive eligibility, intake, and engagement criteria were associated with the inability for Black PWUD to utilize treatment.

You have functioning addicts who may need to work. So am I going to let it all fall apart if I'm going to this treatment center, and I lose my job?…You have to choose: my children or being clean, my job or being clean, eating or [being clean].

### Consequences of The 4Ss

#### Lack of trust in systems and providers

Participants suggested pervasive healthcare and service barriers lead to a lack of trust in systems and providers to provide equitable care to Black PWUD, which in turn prevents Black PWUD from seeking needed services. One peer stated*, “*I believe a lot of people actually would [go to treatment], if they trusted that treatment facility that they weren't going to be mistreated.*”* When Black PWUD do engage with services, participants stated it becomes a *“*re-traumatizing and trust-destroying process,” where they are met without compassion or poor care quality. Participants linked lack of trust to OOD risk via hesitation to engage with needed services.

[There is] a lot of mistreatment at the hospitals when they do go to try to detox before they can go into treatment. And so when you're being mistreated, I mean, you're not going to stay… You're going to obviously leave. And this happens time and time again with people that I'm working with.

The lack of trust also extended to non-healthcare systems as participants again did not distinguish between substance use, health, and public services. One example discussed was Black PWUD being wary of Missouri’s 911 Good Samaritan law [19] designed to protect individuals from prosecution when they call 911 in response to an overdose: “A lot of people just let a person sit there and OD [i.e., overdose] because they feel like they're going to get blamed for them nodding [off].”

#### Drug use as coping

Participants described how a lack of The 4Ss drove drug use and OOD among Black people by providing a respite from their chronic unmet needs.

“To go back to why are [Black] people doing drugs, have you seen what the world looks like for them? What else are they going to do? If you have no source of stability, no safety, nobody that you can go to when you're struggling with impossible situations to back you up and make you feel like you can do this without drugs, then there's no safety for you. At least you can feel safe for a little bit [by using drugs], and get a break from this, because there's no break for most people.”

A lack of 4Ss was thought to significantly impact Black people in early life. Participants identified childhood traumatic experiences like parental drug use and physical and sexual abuse as underlying drug misuse and overdose. When asked what drives people to use drugs, one outreach worker stated: “*Trauma, it begins with trauma. Childhood trauma that goes into adolescent trauma, that turns into adult trauma.*” As such, unmet 4S needs and resulting drug misuse is often intergenerational, leading to hopelessness within Black families.

#### Health and social consequences

Participants described how Black people sell drugs to meet their basic financial needs and gain the security of social support in the drug trade.

I didn't go to school ‘cause I felt like, "I'm not going to be alive when I'm 21. Boy, you want me to spend four years of my life going to this high school? I'm not even going to be alive when I leave here. It's a waste of my time…. So I'm going to sell dope, because…at least I can get something now, and when I die later, at least I had died with a little something more than what I had.”

Some turn to gangs for support, which can lead to involvement in the illegal drug trade, gun violence, and engagement with the criminal justice system. One CHW described how the illegal drug trade helped meet his own 4S needs.

That was my support system. So even when you think about selling drugs, using drugs … I always say it was love, but it was a broken form of love… you end up searching for what you're missing and the people who are right around you is searching for the same thing.

Participants also discussed how bullet-related injury associated with gun violence contributed to poorly managed health consequences. These consequences included opioid dependence and OOD, which participants associated with prescribing practices used to bullet-related injuries: “*I have a [client] who he got shot in his stomach and [the hospital] had him on a fentanyl drip for a month…now it’s to the point where his body’s telling him “You need this [drug] in order to survive.*”

### Strategies for mitigating The 4Ss

#### Cultural strategies for resilience

Participants emphasized the importance of cultural strategies for mitigating the impact of the lack of 4Ss on OOD. Individual protective strategies focused on Black cultural strengths, including racial pride, and religious and faith practices. Participants described the importance cultural strengths for cultivating hope, resilience, and social support in the face of The 4Ss.

When I was a child…we had prayer in the schools. We didn't think nothing of it, but that prayer and that foundation taught us compassion, that taught us to care, told us to reach for one another.

#### Trusted community messengers

Practical strategies included trusted messengers. Participants described how Black PWUD require trusted voices–peers and people from their own communities—to provide accurate education and deliver services that cultivate 4S needs.

That's what I love about [being a CHW]. We are the people from the neighborhood who have gone. We look like them, we've experienced the things that they've experienced, and we see the value in the hood.

Participants perceived that the trust and hope they engendered in clients as members of the same communities–both racially and geographically—made them more effective than traditional healthcare workers. CHWs and peers described the social support they provide as an important mechanism for mitigating The 4Ss for Black individuals and communities and, in turn, for preventing substance misuse and facilitating recovery.

#### Harm reduction information and tools

Peers and CHWs found their ability to engender trust and be mobile through street outreach particularly invaluable for connecting Black PWUD to harm reduction information, education, and safe use tools like naloxone and clean needles.

A lot of people are kind of happy that we started the needle exchange…and then we can educate them on where to exactly stick theyselves…They feel comfortable with me enough to be like, “I don't want to jack myself somewhere where it don't need to be. So if you know where it should go, could you help me?”

### Intervening conditions: fentanyl and COVID-19

The impact of The 4Ss on OOD must be considered in light of a fentanyl-contaminated drug supply. Participants described how their work to reduce OOD among Black people was made difficult by the increasing availability of illicit fentanyl. Participants associated increasing OOD among Black people with fentanyl-contaminated stimulants, particularly cocaine: “I had a friend…he thought it was just pure crack and come to find out it was fentanyl. He OD’d and now he’s in the hospital.” Participants also perceived fentanyl was purposefully and disproportionately distributed in Black neighborhoods due to systemic racism.

How do you think these people are getting this fentanyl? It's not coming from us. It's coming from the big people. They're giving it to these little people out here…Y'all are giving it to the [Black] community. Just like with the crack cocaine.... Y'all gave it to the [Black] community.

As focus groups were conducted in 2021, participants also noted how the COVID-19 pandemic made healthcare and social resources more difficult to access and harm reduction more difficult to practice: “*The pandemic isolating people, even people that I know that religiously carry Narcan and know how to use it, if they're not able to be using with a buddy…or they're trying to stay distant, then it's much more dangerous.*”

## Discussion

The current study examined why rates of OOD were rapidly increasing among Black St. Louisans by drawing upon insights of peer outreach workers and CHWs who both represent and serve this community. The resulting grounded theory suggests unmet basic economic, physical, psychological, and social needs (i.e., The 4Ss) resulting from systemic racism drive OOD. Although trauma exposure and unmet social determinants of health are well-documented drivers of substance use [20, 21], particularly among minoritized racial groups [22], our findings illustrate how specific manifestations of systemic racism, specifically at the neighborhood and healthcare levels, lead to and perpetuate trauma, isolation, and unmet needs. These two contextual themes echo previous scholarship detailing the calamitous effects of historical racism on the Black communities not only via on-average lower socio-economic status, but also “via inequalities in power, prestige, freedom, neighborhood context, and health care” [23].

Given the persistence of racial segregation and related racial inequities in St. Louis, the theory of syndemics [24–26] is a useful framework for interpreting the current grounded theory. Syndemic theory suggests that communities already facing health threats are most impacted by emerging epidemics due to underlying oppression [27]. For example, participants described not only how Black people in St. Louis are disproportionately affected by the opioid crisis, but also interrelated and compounding conditions such as bullet-related injury and COVID-19. Thus, systemic racism and ongoing health inequities interacted with the emergence of illicitly manufactured fentanyl to exacerbate the impact of The 4Ss on OOD among Black St. Louisans. Taken with evidence that Black Americans are less likely than white Americans to receive opiate pain medication and medication for OUD due to systemic and individual racism [28–30], our findings suggest that although unmet 4S needs are not new to Black St. Louisans, they interacted with a racialized shift in opioid availability (i.e., from prescription opioids to fentanyl) to increase OOD risk [31]. Future research should clarify the intervening influence of fentanyl’s ubiquity not only on OOD, but also on Black community strategies to mitigate them (e.g., harm reduction education and uptake).

In particular, the contextual theme of neighborhood disinvestment reflects the racist geographic history of St. Louis. St. Louis remains one of the most segregated cities in the USA [32], with the infamous east–west Delmar Boulevard dividing the region city racially and socioeconomically as an artifact of segregationist policies [33]. Accordingly, participants emphasized the importance of upstream approaches to reducing OOD, suggesting that improving neighborhood amenities such as schools, parks, and grocery stores may reduce OOD and support recovery, which aligns with studies showing neighborhood investment improves social capital and overall well-being [34, 35]. Participants also stressed the importance of increasing treatment access in their communities to address healthcare and service barriers but noted Black PWUD cannot maintain recovery if broader 4S needs remain unmet as drug use is an effective strategy for coping with The 4Ss. These findings echo calls to expand funding for interventions prioritizing basic needs, such as the “Housing First” model, which is associated with increased rates of stable housing, general well-being, and healthcare utilization [36].

Although findings suggest St. Louisans need increased access to services to prevent OOD in Black communities, how those services are delivered determines their effectiveness. Themes related to provider mistrust and the importance of trusted community messengers reflected participants’ resounding perception that care, consistency, and understanding were lacking in traditional service delivery for Black PWUD. This study supports evidence that peers and CHWs can fill this gap by providing safe spaces and culturally responsive care. Previous scholarship demonstrates peer relationships improve recovery outcomes for PWUD [37–39] and help people stay in remission rather than return to drug use, which is thought to be the most important factor in reducing OUD over time [40]. We add that shared racial identity and neighborhood context among peers are key to building trust. In racially segregated Black communities, peer support from neighborhood-based, grassroots organizations may provide a more effective bridge to care than that housed in traditional treatment institutions due to systemic service barriers. More research is needed to clarify institutional and cultural mechanisms of peer service delivery among Black PWUD.

Limitations of this study include the purposive sampling method, which although appropriate for the study aim, likely influenced the findings as many participants were in recovery. However, their individual experiences with drug use, overdose, and treatment largely pre-dated the noted increases in Black OOD. Although participants may have experienced a non-fatal overdose, they were unable to represent those who were unfortunately lost to OOD. Given the community-engagement principles employed, the researchers also had a previous professional relationship with some participants. To mitigate the impact of pre-existing relationships, no participants were involved with CENTER in a professional or advisory capacity. Nonetheless, this could have influenced focus group discussions. Discussions also may have been influenced by the positionality of focus group facilitators, who were both white women. Despite authors, CAB members, and community partners agreeing that saturation was reach, findings are based on a small sample localized to St. Louis, which limits its application.

## Conclusions

Focus groups with key stakeholders suggest interventions promoting basic social, physical, and economic needs are urgently needed to reduce OOD among Black PWUD. Grassroots efforts utilizing peer services can build trust and hope through the provision of culturally congruent interventions and promote harm reduction in Black communities underserved by health and social systems. Future research should leverage the current stakeholder-informed model to examine effective strategies for reducing racial inequities in OUD treatment and sequelae.

## Data Availability

Data sharing is not applicable to this article as no datasets were generated.
